# Increased risk of chronic kidney disease in uric acid stone formers with high neutrophil-to-lymphocyte ratio

**DOI:** 10.1038/s41598-023-45034-1

**Published:** 2023-10-17

**Authors:** Hsiu-Ting Tung, Chia-Min Liu, Ho-Shiang Huang, Ze‐Hong Lu, Chan-Jung Liu

**Affiliations:** Department of Urology, National Cheng Kung University Hospital, College of Medicine, National Cheng Kung University, No. 138, Sheng Li Road, Tainan, 704302 Taiwan

**Keywords:** Nephrology, Urology, Biomarkers, Risk factors

## Abstract

Urolithiasis is associated with an increased risk of chronic kidney disease (CKD), irrespective of stone compositions. Chronic inflammation is an important factor for CKD progression. Neutrophil-to-lymphocyte ratio (NLR) has been recognized as a reliable biomarker of inflammation, yet its use in predicting renal deterioration in patients with urolithiasis remains limited. We aimed to explore whether the combination of stone composition and NLR could be useful as a predictor for CKD risk. A total of 336 stone formers with at least one stone submission for analysis were enrolled in the retrospective study. Stones were classified into uric acid and calcium groups. Renal functions were assessed at least one month after stone treatment. Uric acid stone formers had significantly lower estimated glomerular filtration rate (eGFR) compared with calcium stone formers (*p* < 0.001). NLR was significantly higher in uric acid stone formers (*p* = 0.005), and a significantly negative correlation (*p* < 0.001) between NLR and eGFR had been observed only in uric acid stone group. Univariate and multivariate logistic regression analyses showed that higher proportion of uric acid stone composition and higher NLR were both significantly associated with CKD risks. A nomogram integrating independent predictors was generated for CKD prediction, yielding an AUC of 0.811 (0.764–0.858). In conclusion, our study demonstrated that stone formers with higher proportion of uric acid composition and higher NLR levels were associated with higher CKD risk.

## Introduction

Urolithiasis has been reported to be associated with an increased risk of chronic kidney disease (CKD) and progression to end stage kidney disease (ESKD)^1,2^. The mechanisms by which urolithiasis may lead to CKD remain largely uncertain. Recurrent urinary tract infections (UTI), hydronephrosis secondary to obstruction, or treatments for urolithiasis are plausible contributors to urolithiasis-related CKD^2,3^. One might hypothesize that an increased prevalence of metabolic syndromes such as diabetes mellitus (DM), obesity, dyslipidemia, and hypertension (HTN) could also partially explain the increased risk of CKD among stone formers.

Urolithiasis can be categorized according to stone compositions. Calcium oxalate (CaOx) is the most common type of stones (75–90%)^4^, and the majority of CaOx stones are considered idiopathic^5^. Most calcium phosphate (CaP) stones are possibly associated with UTI, whereas uric acid stones are highly related to metabolic syndromes^6^. The distinct characteristics and mechanisms of formation may have different influence on renal function, but limited research has specifically examined the risk of CKD associated with different stone types. Therefore, it is important to investigate the impacts of specific types of urolithiasis on renal function, adjusted for well-known CKD risk factors.

Chronic inflammation produced in response to the formation of kidney stone have been proposed as a molecular theory of renal damage associated with urolithiasis. Accumulating evidence suggests that systemic inflammatory response can be assessed by neutrophil-to-lymphocyte ratio (NLR), which is a readily available and cost-effective parameter derived from a blood count measurement commonly performed in outpatient and inpatient settings^7^. NLR not only could predict the prognosis of many cancers^8^, but is also associated with the risk of proteinuria, CKD, and ESKD^9–11^. However, in the field of urolithiasis, NLR has only been used to predict postoperative fever^12^ and spontaneous stone passage^13^, but to the best of our knowledge, NLR has not yet been validated as a predictor of urolithiasis-associated CKD.

The objective of this study was to evaluate whether the combination of specific stone composition and NLR could predict the risk of CKD.

## Materials and methods

### Study population and data collection

We conducted a retrospective cohort study by analyzing data of 336 urolithiasis inpatients from a single tertiary medical center from March 2011 to October 2021. All enrolled patients had received endoscopic treatment for the urolithiasis, and the extracted stones were subsequently analyzed for stone composition. The routine follow-up schedule usually included a clinic visit one week after discharge, followed by the removal of the ureter stents and blood tests one month later.

**Figure 1:**
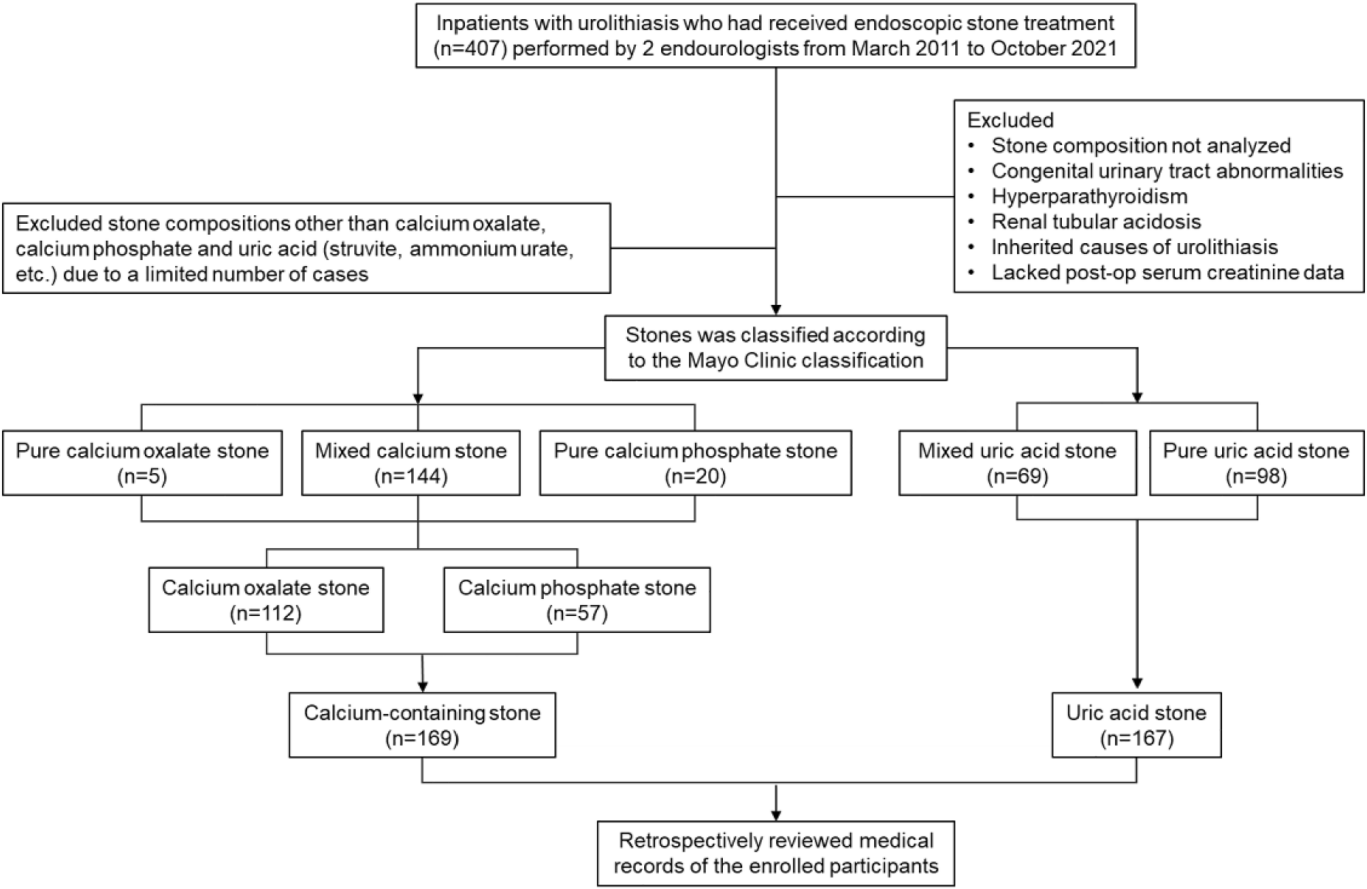
Flow diagram of the cohort study.

Patients with documented history of congenital urinary tract abnormalities, hyperparathyroidism, renal tubular acidosis, or inherited causes of urolithiasis were excluded. Stone analysis was performed using a standard Frontier Fourier transform infrared spectroscopy (FTIR) spectrometer, allowing for precise analysis of complexity of each crystal type. In brief, retrieved stones were cleaned, dried, and stored at room temperature until analyzed. Each sample was crushed, and a small fragment of the stone sample was mixed with 200 mg potassium bromide, powdered, and then pressed into a small tablet, which was analyzed by FTIR spectrophotometer (JASCO Corporation, Tokyo, Japan). Stones were classified according to the Mayo Clinic classification practices^14^. Figure 1 showed the sources and methods of selection of participants, and the classification of stone types. First, all subjects were classified into two groups: non-uric acid containing (calcium-containing) stone group and uric acid stone group. Patients with stones containing any uric acid were placed in the uric acid stone group. Second, we took the percentage of stone composition into analysis. Uric acid stone formers were classified into mixed and pure groups. Calcium-containing stone formers were classified into pure CaOx, pure CaP, and mixed calcium stone groups. Patients with stone compositions other than CaOx, CaP, and uric acid were excluded due to a limited number of cases. The study protocol was reviewed and approved by the institutional review board of National Cheng Kung University Hospital (Registry numbers: A-ER-107-291, A-ER-111-093), and the requirement for informed consent was waived because of the retrospective nature of the study.

Baseline characteristics, including demographic information, comorbidities, stone compositions, and metabolic profiles were retrospectively reviewed. The analyzed variables included age, body mass index (BMI), gender, urine pH level, serum uric acid, serum cholesterol, serum triglyceride, serum HbA1c, and presence of comorbidities such as type 2 diabetes mellitus (DM), hypertension (HTN), dyslipidemia, cardiovascular disease (CVD), and gout. The eGFR was calculated using the 2021 CKD-EPI equation^15^. Overweight was defined as BMI > 25 kg/m^2^ and acidic urine was defined as urine pH < 6. CKD is defined as eGFR < 60 mL/min/1.73 m^2^. Serum creatinine data used to calculate eGFR was collected at least one month after surgery for urolithiasis, excluding cases of acute kidney injury, and data during periods of stable renal function was selected.

### Statistical analysis

Statistical analyses were conducted using IBM^®^ SPSS^®^ Statistics version 25 and GraphPad Prism 8.3.0 with a two-sided approach. Statistical significance was determined at a p-value threshold of 0.05. Continuous variables were presented as mean ± standard deviation or median (interquartile range), while categorical variables were presented as number (%). The Kolmogorov–Smirnov test was employed to test whether variables were normally distributed. Categorical variables were compared between different groups using Fisher's exact test or Chi-square test, whichever was applicable. Continuous variables were compared between different groups using *t* test, one-way analysis of variance (ANOVA), Mann–Whitney–Wilcoxon test, or Kruskal–Wallis test, depending on the number of comparisons and whether the variables followed a normal distribution. The correlation between two variables was analyzed using Spearman’s rank coefficient of correlation. Univariate and multivariate logistic regression analyses were performed to identify potential risk factors associated with CKD. Due to the retrospective nature of our study, there was missing data with varying magnitudes within some variables. To minimize potential bias produced by the listwise deletion, only variables with no missing data were selected for the multivariate analysis. A nomogram was constructed from the multivariable logistic regression prediction model using rms package in R version 4.2.3. Each identified variable was assigned a specific value based on its contribution to the model, and these point values were then used to calculate a total score, which corresponded to a predicted probability of CKD. A receiver operating characteristic (ROC) curve was constructed to evaluate the predictive performance of the multivariate logistic regression model, and the area under the curve (AUC) was calculated as a measure of the discriminatory ability.

## Results:

### Baseline characteristics and clinical data of study population

A total of 336 stone formers were included in the final analysis (Table 1). Among them, 169 patients (50.3%) had calcium containing stones, and 167 patients (49.7%) had uric acid stones. The mean age of the participants was 60.3 years. Patients with uric acid stones were significantly older compared to those with calcium containing stones (*p* < 0.001). The majority of all patients were male (71.7%), and significantly more men submitted uric acid stones than calcium-containing stones.

**Table 1:** Clinical and laboratory characteristics of the study population.

	Total	Calcium stone	Uric acid stone	*P*-value
	(n = 336)	(n = 169)	(n = 167)	
Age (years)	60.3 ± 13.1	56.5 ± 11.7	64.0 ± 13.4	< 0.001
BMI (kg/m^2^)	26.2 ± 4.2	25.8 ± 4.1	26.6 ± 4.3	0.088
Male (%)	241 (71.7%)	98 (58.0%)	143 (85.6%)	< 0.001
Overweight (%)	198 (58.9%)	97 (57.4%)	101 (60.5%)	0.566
DM (%)	107 (31.9%)	47 (27.8%)	60 (35.9%)	0.110
HTN (%)	157 (46.7%)	68 (40.2%)	89 (53.3%)	0.016
Dyslipidemia (%)	97/144 (67.4%)	45/67 (67.2%)	52/77 (67.5%)	0.963
CVD (%)	27/268 (10.1%)	9/102 (8.8%)	18/166 (10.8%)	0.594
Gout (%)	49/268 (18.3%)	7/102 (6.9%)	42/166 (25.3%)	< 0.001
Hyperuricemia (%)	57/138 (41.3%)	11/42 (26.2%)	46/96 (47.9%)	0.017
Acidic urine (%)	122 (36.3%)	37 (21.9%)	85 (50.9%)	< 0.001
eGFR (mL/min/1.73 m^2^)	78.9 ± 40.1	95.0 ± 41.5	62.5 ± 31.1	< 0.001
Serum uric acid (mg/dL)	6.7 ± 1.8 (n = 138)	6.2 ± 1.9 (n = 42)	6.9 ± 1.6 (n = 96)	0.018
Serum CHOL (mg/dL)	177.5 ± 41.0 (n = 143)	180.5 ± 40.0 (n = 67)	175.0 ± 42.0 (n = 76)	0.423
Serum TG (mg/dL)	139.0 ± 81.9 (n = 143)	137.2 ± 86.9 (n = 67)	140.5 ± 77.8 (n = 76)	0.812
Serum HbA1c (%)	6.4 ± 1.4 (n = 106)	6.4 ± 1.4 (n = 27)	6.5 ± 1.4 (n = 79)	0.741
NLR	2.8 (1.9–4.6)	2.54 (1.68–4.27)	3.16 (2.10–4.99)	0.005

Values are expressed as number (%), mean ± standard deviation, or median (interquartile range).

*BMI* body mass index, *DM* type 2 diabetes mellitus, *HTN* hypertension, *CVD* cardiovascular diseases, *CHOL* total cholesterol, *TG* triglyceride, *HbA1c* glycated hemoglobin, *NLR* neutrophil/lymphocyte ratio.

The case numbers of variables with missing data were specifically annotated.

Regarding comorbidities, patients with uric acid stone were more likely to have DM, HTN, CVD, gout, and dyslipidemia, while statistical significance were only reached for HTN (*p* = 0.016) and gout (*p* < 0.001). Laboratory test results revealed that patients with uric acid stone were more likely to have low urinary pH level (*p* < 0.001), as well as hyperuricemia (*p* = 0.017). Notably, renal function was significantly worse in patients with uric acid stone compared with calcium containing stone (*p* < 0.001). In addition, NLR was significantly higher in patients with uric acid stone (*p* = 0.005).

### Stone composition and renal function

**Figure 2:**
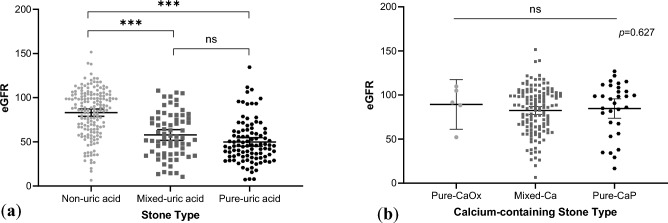
Different stone groups and their eGFR. (**a**) Grouped scatterplot of non-uric acid group, mixed uric-acid stone group, and pure acid stone group and their eGFR; (**b**) Grouped scatterplot of pure calcium oxalate stone group, mixed calcium stone group, and pure calcium phosphate stone group and their eGFR.

Due to the findings that uric acid stone formers had significantly worse renal function than calcium stone formers, we attempted to stratify stone formers based on different proportion of their stone components. First, we studied the renal functions of stone formers with varying percentages of uric acid in stone composition. Patients with pure and mixed uric acid stones had significantly lower eGFR (both *p* < 0.001) compared to those with non-uric acid stones (Fig. 2a), with eGFR values 49.8 ± 24.3, 57.8 ± 25.3, and 83.1 ± 26.9 in patients with pure, mixed, and non-uric acid stones, respectively. Second, we investigated the renal functions of stone formers with different components of calcium-containing stones using the following three groups: pure CaOx, mixed calcium, and pure-CaP. There were no significant differences in renal function (*p* = 0.797) observed between these groups (Fig. 2b), with eGFR values 89.4 ± 22.6, 82.5 ± 26.4, and 84.7 ± 30.0 in patients with pure CaOx, mixed calcium, and pure-CaP, respectively. To sum up, the presence of uric acid component in stone formers is associated with worse renal function, while the association is not observed with the CaOx and CaP components.

### The NLR values and renal function

**Figure 3:**
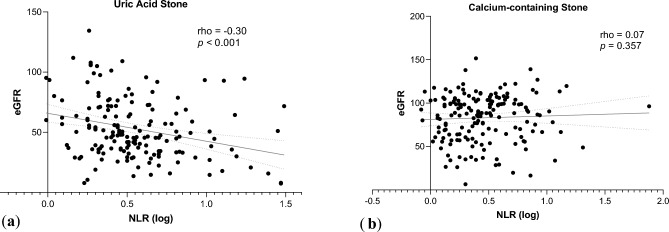
The correlation of eGFR and NLR. (**a**) Scatterplot of log-transformed NLR and eGFR in uric acid stone group; (**b**) Scatterplot of log-transformed NLR and eGFR in calcium stone group.

As shown in Fig. 3a, in uric acid stone group, a Spearman’s rank coefficient of correlation revealed a significantly negative correlation between NLR and eGFR (Spearman’s ρ = − 0.30, *p* < 0.001). In contrast, no significant correlation had been observed between NLR and eGFR (*p* = 0.357) in calcium stone group (Fig. 3b).

Among all participants, the median NLR was 2.8. The study population was further divided into two groups based on a NLR cutoff of 2.8 for subsequent analysis. Participants with an NLR equal to or below 2.8 were classified into Low NLR group, while those with an NLR above 2.8 were classified into High NLR group (Supplementary Table 1).

### Identification of risk factors associated with CKD

**Figure 4:**
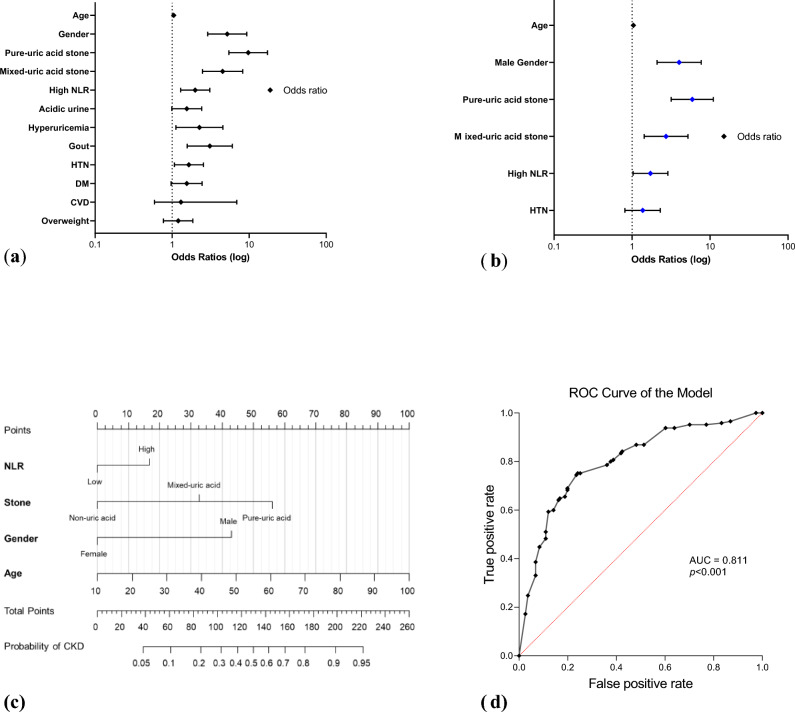
Identification of risk factors for CKD. (**a**) Univariate logistic regression analysis; (**b**) Multivariate logistic regression analysis; (**c**) Nomogram based on the logistic regression model to predict the probability of CKD; (**d**) ROC curve for assessment of the discriminatory ability of the model.

Table 2 showed the results of univariate and multivariate logistic regression analyses. Univariate regression analysis revealed that male gender, age, high proportion of uric acid in stone composition, high NLR, HTN, gout, and hyperuricemia were associated with CKD (Fig. 4a). Multivariate regression analysis was performed to examine the combined effects of the identified variables, which revealed that male gender (*p* < 0.001), age (*p* = 0.002), high proportion of uric acid in stone composition (mixed group, *p* = 0.002; pure group, *p* < 0.001), and high NLR (*p* = 0.039) remained significantly associated with the risk of CKD (Fig. 4b). It is noteworthy that the presence of uric acid stones was associated with a 2.7 to an almost sixfold increase in the risk of CKD. The result of multivariate regression analysis suggested that male gender, older age, uric acid stone composition, and high NLR are independent variables associated with CKD.

**Table 2:** Logistic regression analyses for predicting CKD risks.

	Univariate analysis			Multivariate analysis		
	OR	95% CI	*P* value	OR	95% CI	*P* value
Gender						
Male (vs. female)	5.20	2.90–9.31	< 0.001	4.01	2.09–7.70	< 0.001
Age						
Continuous	1.05	1.03–1.07	< 0.001	1.04	1.01–1.06	0.002
Stone composition						
Non-uric acid	(ref)			(ref)		
Mixed-uric acid	4.53	2.48–8.26	< 0.001	2.73	1.43–5.21	0.002
Pure-uric acid	9.72	5.46–17.29	< 0.001	5.92	3.18–11.02	< 0.001
NLR						
High (> 2.8 vs. ≤ 2.8)	1.99	1.29–3.09	0.002	1.72	1.03–2.88	0.039
Overweight						
Yes (BMI > 25 vs. ≤ 25)	1.20	0.77–1.86	0.426			
DM						
Yes vs. No	1.55	0.97–2.45	0.065			
HTN						
Yes vs. No	1.65	1.07–2.55	0.024			
Gout						
Yes vs. No	3.09	1.57–6.06	0.001	1.37	0.81–2.30	0.242
CVD						
Yes vs. No	1.30	0.59–6.90	0.517			
Hyperuricemia						
Yes (> 7 vs. ≤ 7 mg/dL)	2.26	1.12–4.57	0.023			
Dyslipidemia						
Yes vs. No	0.80	0.40–1.60	0.524			
Acidic urine						
Yes (pH < 6 vs. ≥ 6)	1.55	0.99–2.42	0.056			

Parameters used for multivariate regression analysis: gender, age, stone composition, NLR, HTN. *OR* odds ratio, *CI* confidence interval, *DM* type 2 diabetes mellitus, *HTN* hyper-tension, *CVD* cardiovascular diseases, *NLR* neutrophil/lymphocyte ratio.

### Generation of a nomogram for the logistic regression model

Subsequently, a nomogram integrated all the independent variables was generated, as shown in Fig. 4c, with which patients’ individual probability of CKD can be calculated based on their characteristics. In the nomogram, each variable had a distinct score on the “Points” scale. By summing the scores assigned to all variables, the cumulative score could be located on the “Total points” scale. The probability of CKD could be obtained by drawing a vertical line down to the corresponding “Probability” scale. The nomogram incorporated gender (male, female), age, uric acid stone composition (non-, mixed-, pure-uric acid stone), and high NLR (≦ 2.8, > 2.8) to determine a probability of CKD between 5 and 95%. For example, an 80-year-old female patient with a pure uric acid stone, and an NLR equal to 3.0 has a 70% chance of developing CKD. An ROC analysis was used to assess the discriminatory ability of the model, which revealed an AUC of 0.811 (0.764–0.858, *p* < 0.001), as shown in Fig. 4d. The nomogram could serve as a graphical and straightforward means for providing more accurate and personalized predictions of the risk of CKD.

## Discussion

This retrospective cohort study was performed to demonstrate that a higher NLR, the presence of uric acid stone composition, and a higher uric acid stone composition, are independently associated with a higher risk of developing CKD in stone formers after considering underlying comorbidities and baseline parameters. In addition, the combination of age, gender, uric acid stone composition and NLR can significantly improve the risk stratification for urolithiasis-related CKD, suggesting that NLR and uric acid stone components could serve as easy and practical indicators to identify high-risk CKD patients following stone intervention.

Current evidence has established a close correlation between urolithiasis and impaired renal function, but the mechanism beneath the correlation remains uncertain. While stone-related obstruction, infection, or renal tissue damage are commonly implicated as potential causes, a considerable proportion of patients without these conditions still experience worsening renal function. Consequently, this raises an important question: which kind of stone formers will develop impaired renal function? If we can accurately identify patients at high risk for future CKD, then a proactive kidney stone removal should be encouraged in such populations.

Based on our present findings, the importance of uric acid stone formation in the development of CKD should be emphasized. The mechanisms contributing to uric acid stone formation include acidic urine, increased uricosuria, and decreased urine output, with acidic urine being the most significant factor^16^. In previous experimental studies, a low urine pH had been found to be associated with tubulointerstitial damage secondary to reactive oxygen species (ROS) accumulation, and impairment of renal tubular cell proliferation^17,18^. In the long term, the compensatory increase in acid excretion per nephron to compensate for the loss of nephrons further promotes tubulointerstitial damage and contributes to the progression of CKD. A randomized controlled trial also provided evidence that sodium bicarbonate supplementation effectively delays the progression of CKD by elevating urine pH^19^. However, the causes of the unduly acidic urine in these patients are multifactorial, and the mechanistic pathways leading to renal injury were not fully understood. The acidic urine was found to be attributed to an increased diet-independent acid load to the kidneys, as well as impaired ammoniagenesis and excretion for buffering^20^. An acidic environment facilitates uric acid crystallization in the kidney^20^, which is a pro-inflammatory and cytotoxic event. The crystalline uric acid not only cause renal tubular obstruction, microhematuria and proteinuria, but also trigger M1-like macrophage-related interstitial inflammation, fibrosis, and subsequently granulomatous interstitial nephritis^21,22^, which contribute to CKD progression.

In addition to the effect at a cellular level, the acid urine and uric acid stone formation are closely linked to features of the metabolic syndrome, such as abdominal obesity, hypertension, glucose intolerance and dyslipidemia^6,23^. The specific mechanism was not fully elucidated but seems to be related to insulin resistance and chronic inflammation^24,25^. Taken together, existing evidence suggests that a renal environment promoting uric acid stone formation can be both a consequence and a contributing factor to CKD. Therefore, uric acid stone formation itself can be considered as a predictive factor for CKD.

An observational study conducted by Li et al. revealed that patients with uric acid stones have increased risk of CKD, but the study did not provide detailed information regarding the definition of uric acid stone formers or the percentages of uric acid stone composition^26^. Compared with mixed uric acid stone formers, pure uric acid stone formers are reported to have higher incidences of metabolic syndrome, more acidic urine, and lower ammonium excretion^27^. Hence, we tried to stratify uric acid stone formers using different percentages of uric acid component, compared with CaOx and CaP stone formers, to discover the association with urolithiasis-related CKD, which is the first study to our best knowledge.

Another important finding in our study is that a higher NLR could predict CKD in stone formers, especially in the uric acid stone group. Numerous studies have shown that chronic inflammation contributes to progression of CKD, with CKD patients often exhibiting a state of low-grade inflammation^28^. Various inflammatory markers are associated with many CKD-related complications^29^, but limited research had addressed specifically urolithiasis-related CKD. The NLR serves as a novel measure of inflammation, and its predictive values for renal disease risk, metabolic syndrome, and conditions involving systemic inflammation have been demonstrated^7,9,10^. Neutrophils play a critical role in the immune response by releasing pro-inflammatory cytokines and generating ROS. Neutrophilia is commonly observed in response to inflammatory stimuli, stress, or trauma, and activation of neutrophils could induce lymphopenia through an ROS-dependent mechanisms^30^. The imbalance between neutrophilia and lymphopenia, as indicated by an elevated NLR, suggests a dysregulated immune response and an ongoing systemic inflammation. The normal range of the NLR in healthy adults was proposed to be between 1 and 2, and values higher than 3 are considered pathological, while values between 2 and 3 are considered a grey zone, representing a state of low-grade inflammation^31^. As previously mentioned, the formation of uric acid stones could be considered as a renal manifestation of systemic inflammation^16^. Our study further supports the notion by demonstrating that NLR has a significantly negative correlation with eGFR in uric acid stone group, but not in calcium stone group, implicating that systemic inflammation plays a significant role in uric acid stone-related CKD. Therefore, it is justifiable to incorporate the degree of uric acid stone component and NLR to enhance the predictive capability for CKD. Recently, some novel research had utilized computational methods to predict associations of genetic markers with specific diseases, as well as interactions among various molecules^32–34^. The advancements in computational biology may enhance our understanding of the mechanisms and molecular pathways of uric acid stone-related CKD in the future. Besides, some theoretical modeling studies of gene/protein signaling networks had offered insights for the development of new therapeutic strategies^35,36^, which hold the potential to identify patients at higher risk in early stages and provide timely treatment.

Several limitations should be acknowledged in our study. First, the retrospective design introduces inherent limitations, such as potential selection bias and information bias caused by missing data. Second, the study is cross-sectional in nature, which limits the ability to determine causal relationships. Third, there is a predominance of elderly male participants in our study population, potentially affecting the generalizability of the findings. In addition, the precise cut-off value of NLR has not yet been defined. Larger-scale validation studies are needed to determine the value for better predictive performance. Lastly, whether these patients have good control of their chronic diseases, such as blood pressure or glycemic control, is not recorded, which may introduce potential bias to the observed associations.

In conclusion, our study demonstrated that stone formers with higher degree of uric acid component in their stones and higher NLR levels have increased risk of CKD, and the prediction of CKD risk in stone formers could be improved after incorporating both factors.

### Supplementary Information

Supplementary Information.

## Data Availability

The data that support the findings of this study are available from the corresponding authors upon reasonable request. The data is not publicly available due to privacy and ethical restrictions.
