# Author Correction: Increased risk of chronic kidney disease in uric acid stone formers with high neutrophil-to-lymphocyte ratio

**DOI:** 10.1038/s41598-024-52552-z

**Published:** 2024-01-24

**Authors:** Hsiu-Ting Tung, Chia-Min Liu, Ho-Shiang Huang, Ze-Hong Lu, Chan-Jung Liu

**Affiliations:** Department of Urology, National Cheng Kung University Hospital, College of Medicine, National Cheng Kung University, No. 138, Sheng Li Road, Tainan, 704302 Taiwan

Correction to: *Scientific Reports* 10.1038/s41598-023-45034-1, published online 17 October 2023

The original version of this Article contained an error in the Materials and methods section, under the subheading ‘Study population and data collection’.

“The study protocol was reviewed and approved by the institutional review board of National Cheng Kung University Hospital (Registry number: A-ER-107-291), and the requirement for informed consent was waived because of the retrospective nature of the study.”

now reads:

“The study protocol was reviewed and approved by the institutional review board of National Cheng Kung University Hospital (Registry numbers: A-ER-107-291, A-ER-111-093), and the requirement for informed consent was waived because of the retrospective nature of the study.”

The original Article has been corrected.

