# Supplementary material for: Increased risk of chronic kidney disease in uric acid stone formers with high neutrophil-to-lymphocyte ratio
Source: Sci Rep. 2023 Oct 17;13:17686. doi: 10.1038/s41598-023-45034-1 (PMC10582096; doi:10.1038/s41598-023-45034-1)
Supplement: Supplementary file 1 — Supplementary Information. [file 41598_2023_45034_MOESM1_ESM.pdf]

Title page

Increased risk of chronic kidney disease in uric acid stone formers with high neutrophil-to-lymphocyte ratio

Hsiu-Ting Tung[1], **Chia-Min Liu**[1], **Ho-Shiang Huang**[1], **Ze-Hong Lu**[1, *], **Chan-Jung Liu**[1, *]

[1]Department of Urology, National Cheng Kung University Hospital, College of Medicine, National Cheng Kung University, Tainan, 704302, Taiwan; willy830401@gmail.com (H.-T.T.); cool2423@gmail.com (C,-M,L.); hshuang54@gmail.com (H.-S-H); lthxd@hotmail.com (Z.-H.L.); dragon2043@hotmail.com (C.-J.L.)

*Correspondence: Ze-Hong Lu (lthxd@hotmail.com); Chan-Jung Liu (dragon2043@hotmail.com)

Address: No. 138, Sheng Li Road, Tainan 704302, Taiwan

TEL: 886.6.2353535 ext.5251 FAX: 886.6. 2766179

	High NLR (>2.8) (n=169)	Low NLR (≤2.8) (n=167)	*P*-value
**NLR**	4.58 (3.46-6.88)	1.87 (1.48-2.36)	
**Age (years)**	60.3±14.5	60.2±11.5	0.939
**BMI (kg/m^2)**	26.4±4.5	26.1±3.9	0.451
**Male (%)**	129 (76.3%)	112 (67.1%)	0.059
**Overweight (%)**	100 (59.2%)	98 (58.7%)	0.927
**DM (%)**	60 (35.5%)	47 (28.1%)	0.148
**HTN (%)**	79 (46.7%)	78 (46.7%)	0.994
**Dyslipidemia (%)**	54/86 (62.8%)	43/58 (74.1%)	0.154
**CVD (%)**	13/131 (9.9%)	14/137 (10.2%)	0.936
**Gout (%)**	33/131 (25.2%)	16/137 (11.7%)	0.004
**Hyperuricemia (%)**	27/65 (41.5%)	30/73 (41.1%)	0.958
**Acidic urine (%)**	68 (40.2%)	54 (32.3%)	0.132
**eGFR (mL/min/1.73 m^2)**	75.0±41.1	82.8±38.7	0.073
**Serum uric acid (mg/dL)**	6.8±1.6	6.6±1.9	0.539
**Serum CHOL (mg/dL)**	172.2±41.5	185.4±39.3	0.057
**Serum TG (mg/dL)**	133.7±65.6	147.0±101.8	0.345
**Serum HbA1c (%)**	6.7±1.6	6.2±1.2	0.047

Supplementary Table 1. Clinical characteristics of the stone formers stratified by values below and above the median NLR.

Values are expressed as the number (percent), mean ± standard deviation, or median (interquartile range).
BMI: body mass index; DM: type 2 diabetes mellitus; HTN: hypertension; CVD: cardiovascular diseases; CHOL: total cholesterol; TG: triglyceride; HbA1c: Glycated hemoglobin; NLR: neutrophil/lymphocyte ratio.

	Non-uric acid (n=169)	Mixed-uric-acid (n=69)	Pure-uric acid (n=98)	*P*-value
**Age (years)**	56.5±11.7	63.1±11.5	64.7±13.1	<0.001
**BMI (kg/m^2)**	25.8±4.1	26.5±3.9	26.7±4.6	0.222
**Male (%)**	98 (58.0%)	59 (85.5%)	84 (85.7%)	<0.001
**Overweight (%)**	97 (57.4%)	44 (63.8%)	57 (58.2%)	0.652
**DM (%)**	47 (27.8%)	21 (30.4%)	39 (39.8%)	0.123
**HTN (%)**	68 (40.2%)	40 (58.0%)	49 (50.0%)	0.034
**Dyslipidemia (%)**	45/67 (67.2%)	20/28 (71.4%)	32/49 (65.3%)	0.858
**CVD (%)**	9/102 (8.8%)	3/68 (4.4%)	15/98 (15.3%)	0.063
**Gout (%)**	7/102 (6.9%)	14/68 (20.6%)	28/98 (28.6%)	<0.001
**Hyperuricemia (%)**	11/42 (26.2%)	16/36 (44.4%)	30/60 (50.0%)	0.050
**Acidic urine (%)**	37 (21.9%)	34 (49.3%)	51 (52%)	<0.001
**eGFR (mL/min/1.73 m^2)**	95.0±41.5	67.1±29.9	59.3±31.6	<0.001
**Serum uric acid (mg/dL)**	6.2±1.9	6.9±1.6	7.0±1.7	0.055
**Serum CHOL (mg/dL)**	180.5±40.0	181.3±38.0	177.5±41.0	0.431
**Serum TG (mg/dL)**	137.2±86.9	139.0±92.4	141.4±68.9	0.965
**Serum HbA1c (%)**	6.4±1.4	6.2±1.0	6.6±1.6	0.578
**NLR**	2.54 (1.68-4.27)	2.85 (2.08-4.67)	3.22 (2.18-5.22)	0.012

Supplementary Table 2. Clinical characteristics of the stone formers stratified by proportion of uric acid in the stone composition.

Values are expressed as the number (percent), mean ± standard deviation, or median (interquartile range).

BMI: body mass index; DM: type 2 diabetes mellitus; HTN: hypertension; CVD: cardiovascular diseases; CHOL: total cholesterol; TG: triglyceride; HbA1c: Glycated hemoglobin; NLR: neutrophil/lymphocyte ratio
